# *Notes from the Field:* Outbreak of Norovirus Linked to a Food Establishment — Illinois, November 2022

**DOI:** 10.15585/mmwr.mm7233a4

**Published:** 2023-08-18

**Authors:** Megan N. Hanley, Shana M. Altman, Angie Phillips

**Affiliations:** ^1^Tazewell County Health Department, Tremont, Illinois; ^2^Illinois Department of Public Health.

On November 26, 2022, the Tazewell (Illinois) County Health Department (TCHD) contacted the Illinois Department of Public Health (IDPH) concerning a large acute gastroenteritis outbreak linked to restaurant A in Illinois. TCHD conducted an outbreak investigation with the assistance of IDPH, including a case-control study that identified 317 norovirus infections among respondents (excluding the primary patient) who dined at restaurant A during November 19–26, 2022.

## Investigation and Outcomes

A probable case was defined as the occurrence of diarrhea (three or more loose stools within 24 hours) or vomiting in a person who dined at restaurant A during November 19–26; probable cases with norovirus RNA detected in a stool specimen submitted to the IDPH laboratory were considered confirmed. Notification of the outbreak and requests for information from persons who had dined at restaurant A during November 19–26 were disseminated to the public by TCHD and restaurant A. The press also shared information about the outbreak on November 28, 2022, encouraging all persons who dined at restaurant A to report this to TCHD; after the release of news stories by the press, the number of reported ill persons doubled.

Overall, 317 case-patients (three with confirmed and 314 with probable norovirus infection) and 40 control patients (persons who dined at restaurant A during November 19–26 and did not become ill) were interviewed initially through an online form to identify epidemiologic links and common food exposures. When secondary phone interviews were conducted to confirm the illness onset date, pizza toppings, salad dressings, and condiments consumed, only 268 ill persons and 40 controls participated; the additional 49 ill persons were lost to follow-up. This activity was reviewed by CDC and was conducted consistent with applicable federal law and CDC policy.*

Although the outbreak originated in Tazewell County, ill persons resided in 10 additional Illinois counties and 12 other states; some secondary cases were reported within the households of restaurant patrons and were not included in the total of 317 case-patients. Restaurant patrons shared illness information and menu items consumed, via an online questionnaire and follow-up interview; among the 317 ill persons sharing information through the online questionnaire and phone calls, 268 (85%) participated in the secondary interview; 49 (15%) ill persons were lost to follow-up. Among the 268 interviewed persons with information on illness onset date, symptoms commenced during November 20–28, with 114 (43%) cases occurring on November 24 (Figure). The mean incubation period (interval from dining at restaurant A until symptom onset) was 22 hours (range = 3–45 hours), and the average illness duration was 37 hours (range = 3–96 hours). Nearly one third of cases (32%) occurred in persons aged 20–49 years (range = 6 months–83 years). Signs and symptoms reported by 317 case-patients through the online questionnaire included vomiting (84%), nausea (80%), diarrhea (68%), myalgias (40%), chills (38%), abdominal cramps (26%), and fever (19%). Seven persons were evaluated in an emergency department, and five visited an outpatient health care provider; no hospital admissions or deaths occurred.

**FIGURE:**
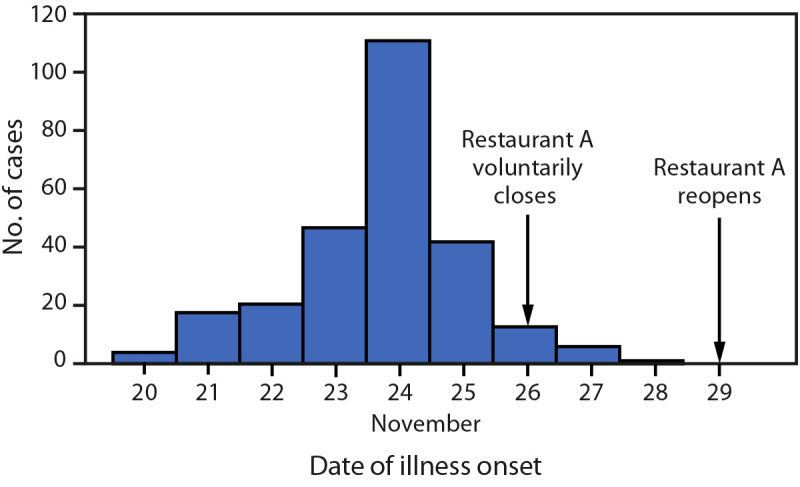
Norovirus cases among restaurant patrons associated with an ill food handler, by date of illness onset (N = 268)* — Illinois,† November 20–28, 2022 <Figure></Figure> * Among 317 total reported cases, onset dates were not included for 49 ill persons with probable cases who were lost to follow-up. ^†^ Although the outbreak originated in Tazewell County, Illinois, ill persons lived in 10 additional Illinois counties and 12 other states.

## Preliminary Conclusions and Actions

Based on data obtained from the questionnaire, the suspected food vehicle was salad (odds ratio = 9.23; 95% CI = 4.48–18.99); 227 of 268 ill persons and 15 of 40 controls consumed salad. Twenty-seven ill persons did not eat salad but did consume additional sauces and dressings. Environmental and epidemiologic investigations indicated that contamination occurred throughout the food preparation process includes the division of the salad, toppings, and dressings into individual portions that are refrigerated for consumption on the following day. Preparation with ungloved hands by a food handler who had vomiting on November 22, and worked during November 21–23, likely served as a main contributor to the outbreak. The restaurant voluntarily closed on November 26 for disinfection and reopened on November 29, after a health inspection. TCHD provided education to food handlers on hand hygiene, staying home from work when ill with diarrhea or vomiting, and cleaning procedures.

Noroviruses are a leading cause of reported foodborne disease outbreaks associated with contamination of food in restaurants during preparation by infected food workers (*1*). A large restaurant-associated norovirus outbreak occurred through the main food vehicle of salad, likely following ungloved hand contact with the salad by an ill food handler during food preparation. Because a large number of persons had patronized the restaurant over the Thanksgiving holiday, the ability to identify exact numbers of ill and well patrons was limited, and the number of cases is likely underreported.

The Food and Drug Administration’s 2022 Food Code cites noroviruses as the leading cause of foodborne illness in the United States, and proper hand hygiene and exclusion of symptomatic employees are essential for preventing outbreaks^†^ (*1*–*5*). Prevention or mitigation of future norovirus outbreaks in food service establishments depends upon reinforcing the need for proper handwashing, performing thorough environmental cleaning, using appropriate personal protective equipment, and excluding workers from the workplace when they are ill with vomiting and diarrhea and for at least 48 hours after resolution of symptoms (*2**–**4*).

## Footnotes

* 45 C.F.R. part 46.102(l)(2), 21 C.F.R. part 56; 42 U.S.C. Sect. 241(d); 5 U.S.C. Sect. 552a; 44 U.S.C. Sect. 3501 et seq.

†
https://www.fda.gov/food/fda-food-code/food-code-2022

## References

[1] Hall AJ, Wikswo ME, Pringle K, Gould LH, Parashar UD; Division of Viral Diseases, National Center for Immunization and Respiratory Diseases, CDC. Vital signs: foodborne norovirus outbreaks—United States, 2009–2012. MMWR Morb Mortal Wkly Rep 2014;63:491–5. 24898166 PMC5779359

[2] Randazzo W, D’Souza DH, Sanchez G. Norovirus: the burden of the unknown. Adv Food Nutr Res 2018;86:13–53. 10.1016/bs.afnr.2018.02.005 30077220

[3] Fumian TM, Ferreira FC, de Andrade JDSR, Norovirus foodborne outbreak associated with the consumption of ice pop, Southern Brazil, 2020. Food Environ Virol 2021;13:553–9. 10.1007/s12560-021-09495-9 34351587

[4] Barclay L, Davis T, Vinjé J. Rare norovirus GIV foodborne outbreak, Wisconsin, USA. Emerg Infect Dis 2021;27:1151–4. 10.3201/eid2704.204521 33754999 PMC8007321

[5] Brennan J, Cavallo SJ, Garman K, Notes from the field: multiple modes of transmission during a Thanksgiving Day norovirus outbreak—Tennessee, 2017. MMWR Morb Mortal Wkly Rep 2018;67:1300–1. 10.15585/mmwr.mm6746a4 30468435 PMC6289081

